# The Study of Antioxidant Components in Grape Seeds

**DOI:** 10.3390/molecules25163736

**Published:** 2020-08-15

**Authors:** Lenka Sochorova, Bozena Prusova, Tunde Jurikova, Jiri Mlcek, Anna Adamkova, Mojmir Baron, Jiri Sochor

**Affiliations:** 1 Department of Viticulture and Enology, Mendel University in Brno, Faculty of Horticulture, Valtická 337, 691 44 Lednice, Czech Republic; tomaskova.l.9@gmail.com (L.S.); bozena.prusova@mendelu.cz (B.P.); mojmir.baron@mendelu.cz (M.B.); 2 Institute for Teacher Training, Faculty of Central European Studies, Constantine the Philosopher University in Nitra, Drazovska 4, Nitra SK-949 74, Slovakia; tjurikova@ukf.sk; 3 Department of Food Analysis and Chemistry, Faculty of Technology, Tomas Bata University in Zlin, Vavrečkova 275, 760 01 Zlin, Czech Republic; mlcek@ft.utb.cz (J.M.); aadamkova@utb.cz (A.A.)

**Keywords:** grape seeds, antioxidant activity, polyphenolic compounds, HPLC/UV-VIS

## Abstract

The paper deals with the study of antioxidant properties of extracts from vine seeds (*Vitis vinifera* L.) using spectrometric and chromatographic techniques. Ten vine varieties (Cerason, Laurot, Kofranka, Gewürztraminer, Hibernal, Blaufrankisch, Zweigeltrebe, Erilon, Palava, and Welschriesling) obtained from the years 2015, 2016, and 2017 were selected for the study. The antioxidant activity was determined spectrophotometrically using four fundamentally different methods; the content of total polyphenolic compounds was determined using the Folin–Ciocalteu method. In 2015, the content of 14 antioxidants (gallic acid, caffeic acid, coumaric acid, coutaric acid, ferulic acid, fertaric acid, trans-piceid, trans-piceatannol, rutin, quercetin-3-β-d-glucoside, quercitrin, myricetin, catechin, and epicatechin) were studied. The results of the study show the high content of antioxidant components in grape seeds and the differences in content in individual varieties and in individual years.

## 1. Introduction

Wine is an important source of phenolic substances, but there are many studies focused on the content of polyphenols not only in wine or grapevine, but also in byproducts of winemaking industry, such as grape seeds or grape stems [1,2,3].

Vine seeds contain a significant source of polyphenolic substances (20–55%), which are important mainly in the production of red wines [4]. Phenolic compounds can be defined as molecules containing a hydroxyl functional group (–OH) attached directly to a benzene ring (aromatic nucleus). Phenolic compounds in berries can be divided into non-flavonoids (single C6 chain, hydroxybenzoic acids, hydroxycinnamic acids, volatile phenols, and stilbenes) and flavonoids (flavones, flavonols, flavanones, flavan-3-ols, and anthocyanins). Non-flavonoid phenols are found in berries and wine. With the exception of hydroxycinnamic acids, they are present in low concentrations [5,6].

Flavonoids form a substantial part of the phenolic compounds present in grapes and include several classes [5,6]. They are C6–C3–C6 polyphenolic compounds containing two hydroxylated benzene rings; A and B are linked by a tricarbon chain that is part of the heterocyclic ring C. According to the oxidation state of ring C, these compounds are divided into structural classes, which include flavonols, flavan-3-ols (including simple flavan-3-ol and their polymeric form, proanthocyanidins), and anthocyanins [7].

The solid waste generated by the wine industry is about 30% total weight and consists mainly of grape marc (containing seeds, pulp, stems and hides). Large amounts of valuable compounds, such as dietary fiber, seed oil, anthocyanins, and phenolic compounds, remain inside the marc [8]. Phenolic compounds have great potential due to their antioxidant properties and can lead to health benefits for and prevention of a variety of conditions, such as coronary heart disease [9], cancer [10], diabetes [11], and neurodegenerative disease [12].

The usage of this plant material as a source of bioactive polyphenolic compounds promises an opportunity to obtain added-value products from winemaking byproducts and merits the attention of the food/pharma industries to provide valuable quality bioactive extracts and innovative added-value co-products [3]. In recent years, the interest in these bioactive compounds has emerged strongly because of the necessity to restrict or reduce the use of synthetic antioxidants addressed to health care and food protection with possible undesirable effects on human and animal health [2,13].

The search of new added-value applications to these byproducts requires the performance of an additional and accurate assessment of its polyphenol profile and the antioxidant capacity of the extracts. This will allow their employment by the food/pharma industry [14].

Antioxidants represent a large and diverse group of substances which work on different principles; therefore, the methodology and possibilities of their determination are often complex. For this reason, the aim of this study was to determine in more detail the polyphenol and antioxidant profile of selected grapevine varieties using a chromatographic method and different spectrometric methods. The novelty of this study is the comparison of interspecific varieties with common grapevine varieties within three years.

## 2. Results

### 2.1. Determination of Antioxidant Activity Using DPPH, FRAP, ABTS, and CHFR Methods

The graphs below show the results of antioxidant activity and content of polyphenolic compounds during the years 2015–2017 in ten vine varieties. These are the average values from the three measurements.

**Figure 1:**
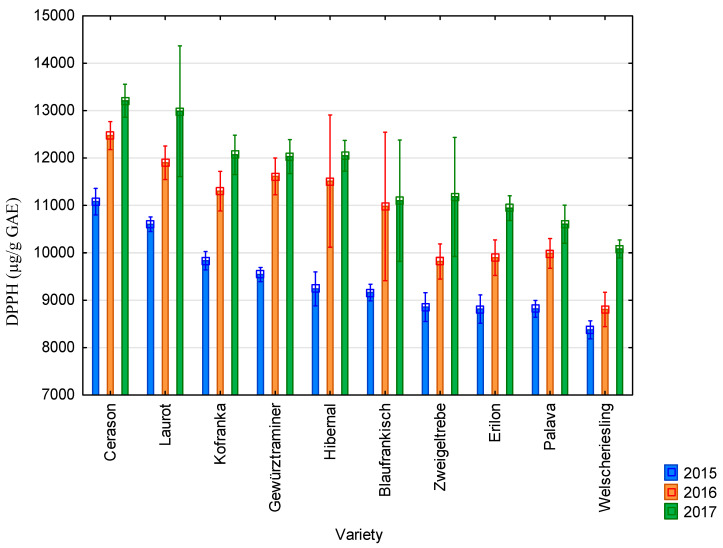
Values of antioxidant activity determined using the DPPH method for individual vine varieties in 2015 (blue bars), 2016 (orange bars), and 2017 (green bars). 2015: *p* < 0.01, 2016: *p* < 0.01, 2017: *p* < 0.01.

The average value of antioxidant activity determined using the DPPH method was 9432 µg/g GAE in 2015; 10,828 µg/g GAE in 2016, and 11,624 µg/g GAE in 2017. All results are presented in Figure 1. The highest values of antioxidant activity studied using the DPPH method were recorded for the Cerason variety in all monitored years (11,079 µg/g GAE in 2015; 12,473 µg/g GAE in 2016; and 13,208 µg/g GAE in 2017). In contrast, the lowest values were recorded for the Riesling variety in all monitored years (8374 µg/g GAE in 2015; 8805 µg/g GAE in 2016; and 10,081 µg/g GAE in 2017). Furthermore, the highest values of antioxidant activity were reached in 2017 and the lowest in 2015. Seeds from blue varieties showed higher values than seeds from white varieties.

**Figure 2:**
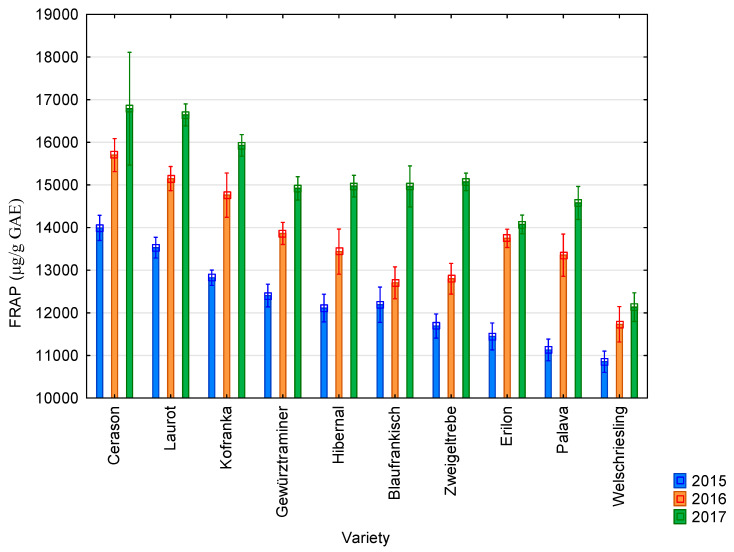
Values of antioxidant activity obtained using the FRAP method for individual vine varieties in 2015 (blue bars), 2016 (orange bars), and 2017 (green bars). 2015: *p* < 0.01, 2016: *p* < 0.01, 2017: *p* < 0.01.

The average value of antioxidant activity determined using the FRAP method was 12,217 µg/g GAE in 2015; 13,724 µg/g GAE in 2016; and 14,807 µg/g GAE in 2017. All results are presented in Figure 2. The highest values of antioxidant activity studied using the FRAP method were recorded for the Cerason variety in all monitored years (13,992 µg/g GAE in 2015; 15,700 µg/g GAE in 2016; and 14,788 µg/g GAE in 2017). The lowest values were recorded for the Riesling variety in all monitored years (10,852 µg/g GAE in 2015; 11,730 µg/g GAE in 2016; and 12,133 µg/g GAE in 2017). The highest values of antioxidant activity were reached in 2017 and the lowest in 2015. Seeds from blue varieties showed higher values than seeds from white varieties.

**Figure 3:**
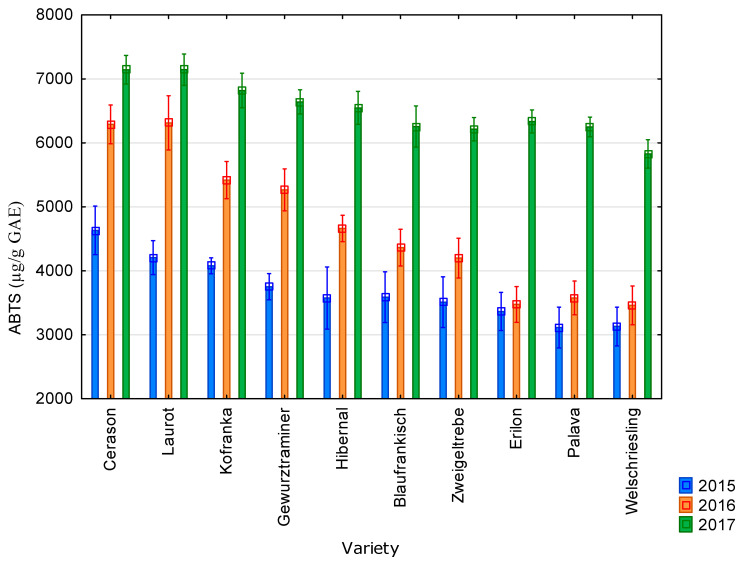
Values of antioxidant activity obtained using the ABTS method for individual vine varieties in 2015 (blue bars), 2016 (orange bars), and 2017 (green bars). 2015: *p* < 0.01, 2016: *p* < 0.01, 2017: *p* < 0.01.

The average value of antioxidant activity determined using the ABTS method was 3695 µg/g GAE in 2015; 4702 µg/g GAE in 2016; and 6518 µg/g GAE in 2017. All results are presented in Figure 3. The highest values of antioxidant activity measured by the ABTS method were recorded for the Cerason and Laurot varieties (4632 µg/g GAE for the Cerason variety in 2015; 6313 µg/g GAE for the Laurot variety in 2016; and 7415 µg/g GAE for the Laurot variety in 2017). In contrast, the lowest values were recorded for the Palava and Welschriesling varieties (3113 µg/g GAE for the Palava variety in 2015; 3460 µg/g GAE for the Welschriesling variety in 2016; and 5828 µg/g GAE for the Welschriesling variety in 2017). The highest values of antioxidant activity were reached in 2017 and the lowest in 2015. Seeds from blue varieties showed higher values than seeds from white varieties.

**Figure 4:**
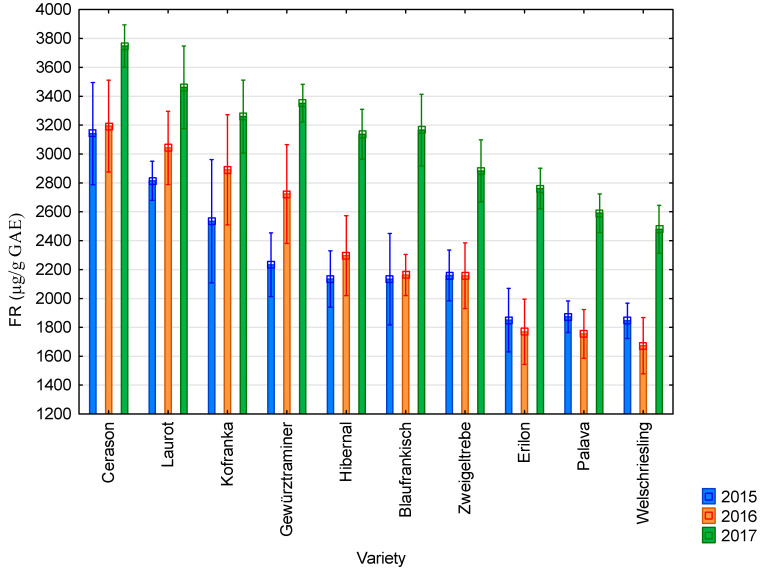
Values of antioxidant activity obtained using the chlorophyllin free radical method for individual vine varieties in 2015 (blue bars), 2016 (orange bars), and 2017 (green bars).2015: *p* < 0.01, 2016: *p* < 0.01, 2017: *p* < 0.01.

The average value of antioxidant activity determined using the chlorophyllin free radical method was 2272 µg/g GAE in 2015; 2366 µg/g GAE in 2016; and 3084 µg/g ^1^ GAE in 2017. All results are presented in Figure 4. The highest values of antioxidant activity studied using the chlorophyllin free radical method were recorded for the Cerason variety in all monitored years (3142 µg/g GAE in 2015; 3193 µg/g GAE in 2016; and 3748 µg/g GAE in 2017). The lowest values were recorded for the Welschriesling in all monitored years (1845 µg.g^−1^ GAE in 2015; 1673 µg/g GAE in 2016; and 2479 µg/g GAE in 2017). The highest values of antioxidant activity were reached in 2017 and the lowest in 2015. Seeds from blue varieties showed higher values than seeds from white varieties.

### 2.2. Determination of Total Polyphenol Concentration

**Figure 5:**
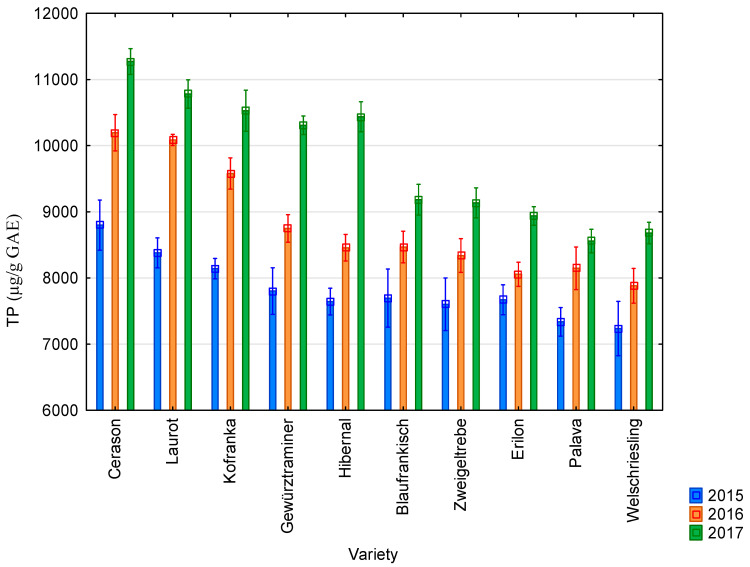
Values of the content of total polyphenolic compounds (TP) for individual vine varieties in 2015 (blue bars), 2016 (orange bars), and 2017 (green bars). 2015: *p* < 0.01, 2016: *p* < 0.01, 2017: *p* < 0.01.

The average value of the content of total polyphenolic compounds was 7831 µg/g GAE in 2015; 8796 µg/g GAE in 2016; and 9782 µg/g GAE in 2017. All results are presented in Figure 5. The highest values of the content of total polyphenolic compounds were recorded for the Cerason variety in all monitored years (8799 µg/g GAE in 2015; 10,196 µg/g GAE in 2016; and 11,272 µg/g GAE in 2017). The lowest values were recorded for the Palava and Welschriesling varieties (7236 µg/g GAE for the Welschriesling variety in 2015; 7882 µg/g GAE for the Riesling variety in 2016; and 8558 µg/g GAE for the Palava variety in 2017). The highest values of the content of total polyphenolic compounds were reached in 2017 and the lowest in 2015. Seeds from blue varieties showed higher values than seeds from white varieties. 

The Pearson correlation coefficient was used to measure the strength of a linear association between two methods for each year. The value *r* = 1 means a perfect positive correlation and the value *r* = −1 means a perfect negative correlation. Results are shown in Table 1.

The values of correlation coefficients from the years 2015–2017 are in the range from 0.79 to 0.97, which shows a positive correlation between the individual methods. The highest values of the Pearson correlation coefficient were 0.97 between the FRAP and DPPH methods in 2015 and between CHFR and ABTS in 2016. In 2017, the highest value of the coefficient 0.92 was between the ABTS and FRAP methods and between TP and CHFR. The lowest value of the coefficient 0.79 was between the FRAP and DPPH methods in 2016 and between the TP and FRAP methods.

### 2.3. Determination of Individual Antioxidant Components by HPLC-UV/VIS

**Figure 6:**
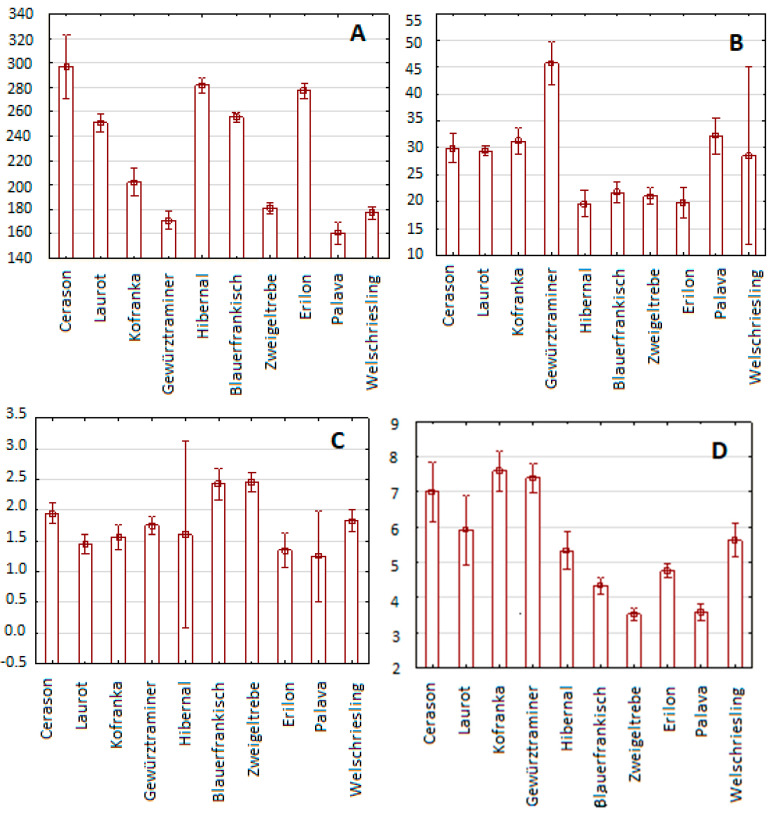

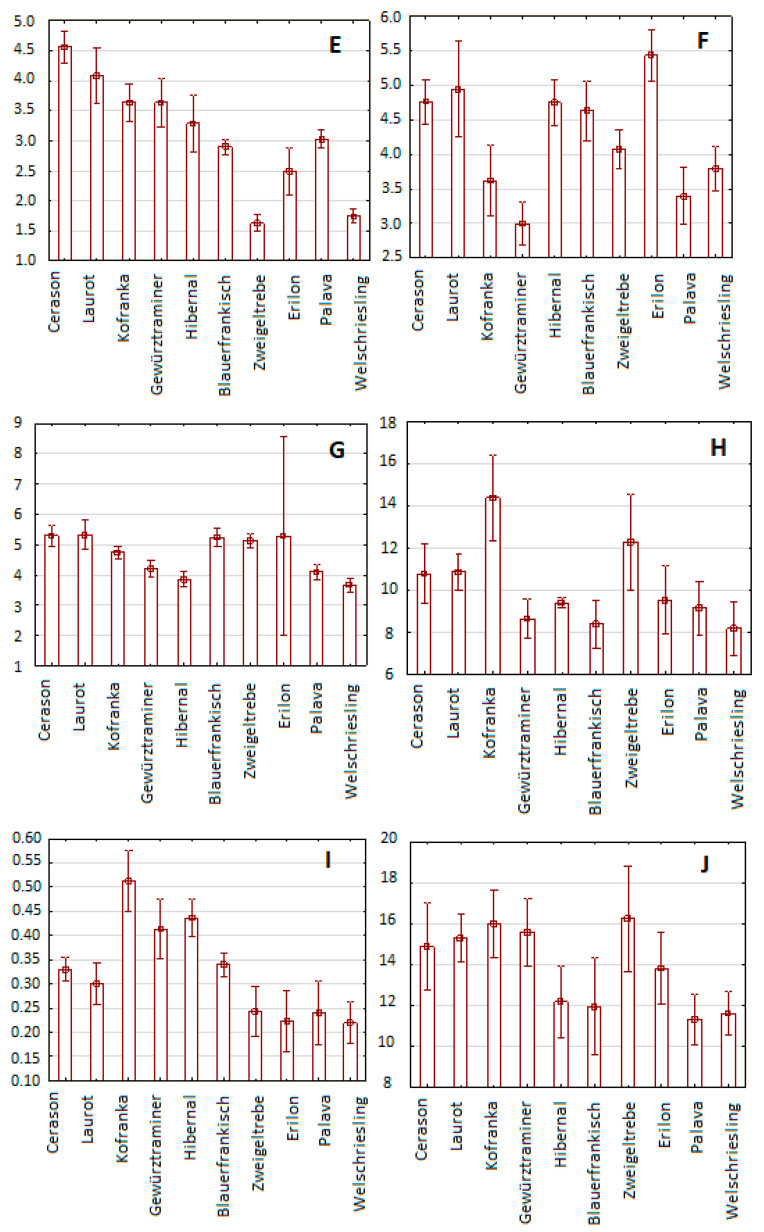

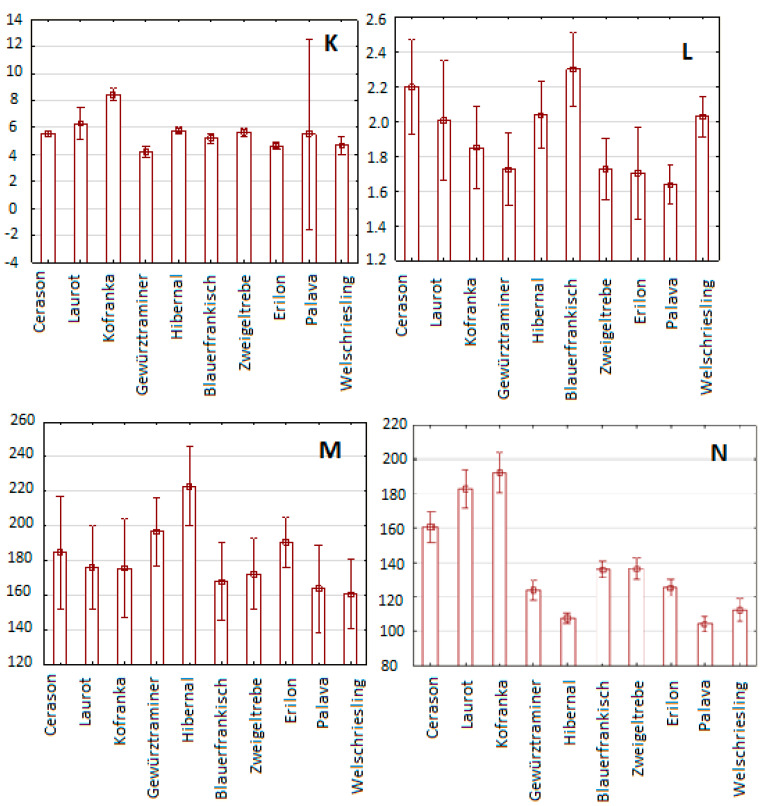
Content of selected antioxidant components in individual grape varieties: gallic acid (**A**), caffeic acid (**B**), coumaric acid (**C**), coutaric acid (**D**), ferulic acid (**E**), fertaric acid (**F**), trans-piceid (**G**), trans-piceatannol (**H**), rutin (**I**), quercetin-3-β-D-glucoside (**J**), quercitrin (**K**), myricetin (**L**), catechin (**M**), and epicatechin (**N**). Results are expressed in µg/g of dry matter.

Using HPLC with UV-VIS detection, the content of selected antioxidant components was determined in the 2015 samples. Attention was focused on the content of gallic acid, caffeic acid, coumaric acid, coutaric acid, ferulic acid, fertaric acid, quercetin, trans-piceid, trans-piceatannol, rutin, quercetin-3-β-D-glucoside, quercitrin, myricetin, catechin, and epicatechin. The graphs below (Figure 6) show the results of the content of these compounds in the individual vine varieties.

Significance levels within individual years of gallic acid: *p* < 0.01, caffeic acid: *p* < 0.01, coumaric acid: *p* < 0.01, coutaric acid: *p* < 0.01, ferulic acid: *p* < 0.01, fertaric acid: *p* < 0.01, trans-piceid: *p* < 0.01, trans-piceatannol: *p* < 0.01, rutin: *p* < 0.01, quercetin-3-β-D-glucoside: *p* < 0.05, quercitrin: *p* < 0.01, myricetin: *p* < 0.05, catechin: *p* < 0.01, epicatechin: *p* < 0.01.

The most represented compound of the monitored antioxidants was gallic acid; its average content in all monitored varieties was 225.4 µg/g. It was present at the highest levels in the Cerason variety (298 µg/g) and was least represented in the Palava variety (160 µg/g ). The second most common compound was epicatechin, with an average content of 138.2 µg/g. The Kofranka variety showed the highest average content of epicatechin: 193 µg/g. The lowest epicatechin content, 107 µg/g, was determined in the Palava variety. Average concentrations of all phenolic compound are presented in Table 2.

The least represented compound was rutin, which had an average content of 0.326 µg/g. The highest content was recorded in the Kofranka variety (0.51 µg/g) and the lowest in the Palava variety (0.22 µg/g). The second least represented compound was coumaric acid; its average value across all monitored varieties was 1.76 µg/g. Its highest content was recorded in the Blaufrankisch variety (2.42 µg/g) and the lowest in the Erilon variety (1.25 µg/g). From these results, it is again clear that seeds from blue varieties showed higher values of antioxidant components than seeds from white varieties.

For ease of comparison with other studies, the results determined by HPLC with UV-VIS for the Cerason variety were used; the values of antioxidant components from 2015 to 2017 were averaged and are summarized in Table 3.

## 3. Discussion

### 3.1. Determination of Individual Antioxidant Components by HPLC-UV/VIS

In a 2016 study by Farhadi et al. [15], polyphenolic substances were determined for six vine varieties; the study examined the content of gallic acid, catechin, epicatechin, caffeic acid, rutin, resveratrol, and quercetin as well as the content of total anthocyanins. Catechin and epicatechin showed the highest measured values, specifically in the blue grapevine variety Ghara Shani, which is typical of western Azerbaijan; its catechin content was 156 µg/g and its epicatechin content was 167 µg/g in grape seed extract. In our study, the highest concentration of catechin 222.5 µg/g was determined in white variety Hibernal and 189 µg/g in Gewürztraminer. The lowest concentration of catechin was determined also in white variety Welschriesling 161 µg/g. The concentration of the epicatechin 192 µg/g was determined in blue variety Kofranka and 183 µg/g in blue variety Laurot. The lowest concentration of caffeic acid in study by Farhadi et al. [15] was determined in Ghara Ghandome variety with a value of 7 µg/g. In our study, concentration of caffeic acid was determined in range 46 µg/g (Gewürztraminer) to 19 µg/g (Hibernal). The concentration of gallic acid was in range 67 µg/g to 91 µg/g, but in our study, it was the most abundant phenolic compound with an average concentration of 225.4 µg/g. 

An experiment by Rockenbach et al. [16] used HPLC to identify anthocyanins, rutin, quercetin derivatives, kaempferol derivatives, catechin, epicatechin, trans-resveratrol, and chlorogenic acid in grape pomace. Catechin was dominant in seed extracts, the highest values of which were recorded in the Cabernet Sauvignon variety with a value of 884.5 µg/g dry matter. Epicatechin was detected only in Pinot Noir (475.0 µg/g dry matter) and Isabella (177.8 µg/g dry matter) varieties. Trans-resveratrol was detected in seeds of the Isabella variety (37.5 µg/g dry matter). Chlorogenic acid was most common in the peel extract of the Isabella variety (231.1 µg/g). Quercetin derivates were not detected.

Research by Guendez et al. [17] examined ethyl acetate extracts of seeds from nine varieties of *Vitis vinifera* L. grown in Greece. They were tested for their content of characteristic polyphenols. The compounds found were major low molecular weight components, including gallic acid, catechin, epicatechin, epigallocatechin, epicatechin gallate, epigallocatechin gallate, and procyanidins B1 and B2. The total content of polyphenolic compounds ranged from 55.1 to 964 µg/g of seeds, with an average of 380 µg/g. The most abundant polyphenol was catechin, which accounted for 49.8% of the total content, followed by epicatechin (26.0%), epicatechin gallate (9.3%), procyanidin B1 (5.8%), and procyanidin B2 (5.1%). Epigallocatechin and galactic acid contents were minor [17]. The average concentration of individual polyphenols in our study ranged from 0.33 µg/g (rutin) to 225.4 µg/g (gallic acid). Total polyphenols concentration was higher, in range 7236 µg/g ^1^ (Welschriesling 2015) to 11,272 µg/g Cerason 2017).

Doshi et al. [18] analyzed phenolic compounds from grape marc (*Vitis vinifera* L.). The study examined the phenolic antioxidants of extracts prepared from the seeds, husks, and tufts of Pusa Navarang and Merlot varieties. HPLC was used to determine the catechin (14,909 µg/g) and epicatechin (9299 µg/g) content of the Pusa Navarang seeds, more higher than in our study, where the catechin concentration was only 181.3 µg/g and epicatechin concentration was 138.2 µg/g of dry matter.

In a study by Hassanpour et al. [19], the content of selected antioxidant components was analyzed using HPLC. Their values were: gallic acid (0.28–3.56 mg/g), catechin (10.02–25.89 mg/g), epicatechin (1.01–17.93 mg/g), chlorogenic acid (2.90–9.82 mg/g), and p-coumaric acid (0.97–5.13 mg/g) [20].

Yilmaz et al. [20] focused on the content of catechin, epicatechin, and gallic acid in the seeds and skins of Merlot, Chardonnay, and Muscadine grapes. Concentrations of epicatechin gallate (ECG), monomeric catechin (MK), and epicatechin (E), all converted to dry matter, were: 99 (ECG), 12 (MK), and 96 (E) µg/g dry matter in Muscadine seeds; 15 (ECG), 358 (MK), and 421 (E) µg/g in Chardonnay seeds; and 10 (ECG), 127 (MK), and 115 (E) µg/g in Merlot seeds. Concentrations of these three compounds were lower in grape skins than in seeds. The results also show that dimeric, trimeric, oligomeric, or polymeric procyanidins represent most of the antioxidant capacity of grape seeds [20]. 

Garcia-Jares et al. [21] used LC/MS/MS to monitor the content of selected antioxidant components in the seeds of white vine varieties. The most common compounds were catechin, epicatechin, and cyanidins B1 and B3. Some compounds (gallic acid, catechin, epicatechin, quercetin-3-glucoside) showed up to several-fold differences in their content between individual varieties. The higher concentration of gallic acid was in Chardonnay (829 µg/g) and the lower in Pinot Gris (296 µg/g). Gewürztraminer contained 296 µg/g of gallic acid; in our study, the seeds of this variety contained 170 µg/g gallic acid. Welschriesling contained 230 µg/g of gallic acid—in our study, 179 µg/g of gallic acid. The most abundant polyphenolic compound was catechin. Its concentration varied from 23,091 µg/g (Pinot Blanc) to 6235 µg/g (Welschriesling) [21]. The average concentration of catechin in our study was only 181.3 µg/g and the higher concentration was found in Hibernal (221 µg/g) and Gewürztraminer (198 µg/g).

### 3.2. Antioxidant Activity and Total Polyphenol Concentration

The results of our study show that the highest concentration of polyphenols in the Cerason variety ranged from 8799 µg/g ^1^ to 11,272 µg/g GAE; this variety also reached the highest AOX values, measured by different methods. A study by Guendez et al. [17] assessed in vitro antiradical activity using the stable DPPH radical. It established a significant correlation between the values of antioxidant activity and the total content of polyphenols (*r*^2^ = 0.6499, *p* < 0.01). However, correlations between the individual compounds showed that procyanidin B1 may be one of the most important scavengers of radicals in grape extracts (*r*^2^ = 0.7934, *p* < 0.002) despite its small share in the total polyphenol content [17]. 

A study by Rockenbach et al. [16] determined the content of total phenols in grape skin and seeds of blue varieties. The highest values of these compounds in seeds were found in the Pinot Noir variety (16,518 µg/g) and the lowest concentration was found in Isabella variety (2128 µg/g). The results were expressed as the equivalent of catechin content (CE). The lowest values of total phenols in our study was found in white varieties Palava and Welschriesling (7236 µg/g for the Welschriesling variety in 2015 and 8558 µg/g for the Palava variety in 2017). Results were expressed as the equivalent of gallic acid (GAE).

In a study by Doshi et al. [18], the antioxidant activity of the Pusa Navarang variety was determined using the FRAP, ABTS, and DPPH methods. The antioxidant activity was 134.8 mg/mL quercetin equivalent (FRAP), 18.7 mM Trolox equivalent (TE) (ABTS), and 33.5 mM Trolox equivalent (DPPH). The content of total phenolic substances was 95.8 mg/mL, content of flavonoids was 30.5 mg/mL, and content of flavan-3-ols was 21.8 mg/mL. 

In a 2017 study, Hassanpour et al. [19] examined the content of polyphenolic compounds and antioxidant activity in the seeds of 20 grapes originating from Iran. The antioxidant activity of the seeds was evaluated using DPPH (final values in the range of 27.34 to 78.57 µmol TEg) and FRAP (final values in the range of 198.83 to 590.86 µmol TEg). The total content of phenolic substances (44.86 to 155.1 mg/g), the total content of flavonoids (23.21 to 131.91 mg/g) and the total values of proanthocyanidins (10.11 to 26.10 mg/g) were also studied. All extracts showed high antioxidant activity.

In a study by Özcan et al. [22], phenolic compounds, minerals, total flavonoids, total phenolic substances, and antioxidant effects of the husks and seeds of table grape varieties were determined. The antioxidant activity in the seeds ranged from 86.688 to 90.974%; the content of polyphenolic compounds was between 421.6 and 490.6 mg GAE/100 g. All results corresponded to the results obtained in our study.

In an extensive study by Rockenbach et al. [16], polyphenolic compounds and antioxidant activity of grape seeds and skins from *Vitis vinifera* L. and *Vitis labrusca* from Brazilian viticulture were studied in Pinot Noir, Isabella, and Cabernet Sauvignon. The seeds had a higher concentration of phenolic compounds (2128 to 16,518 µg/g catechin equivalents (CE)) than the concentration in the husks (660 to 1839 µg.g^−1^ (CE)). The highest values of antioxidant activity, determined using the DPPH radical and the FRAP method, were found in the seeds of the Pinot Noir variety (DPPH: 16,925 μmol TE/100 g and FRAP: 21,492 μmol Fe_2_/100 g) and in the peel extracts of the Isabella variety (DPPH: 3640 μmol TE/100 g and FRAP: 4362 μmol Fe_2_/100 g). 

Jayaprakasha et al. [23] evaluated the antioxidant activity of grape seeds in acetone, ethanol, and methanol extracts using the linoleic acid peroxidation method. At a concentration of 100 ppm, the extracts showed 65–90% antioxidant activity. Extraction with methanol gave the maximum yield of antioxidants, followed by the yield from acetone. The lowest yield was recorded in the extraction with ethanol. 

Antioxidant activity (determined using the DPPH method) and the content of total polyphenols were analyzed in the extracts from the seeds of selected white varieties (Gewürzetraminer, Pinot Gris, Chardonnay, Welschriesling, and Pinot Blanc). The values of antioxidant activity ranged from 31.0 to 41.3 mmol Trolox/g dry matter. The content of total polyphenolic compounds values corresponded to the study by Garcia-Jares et al. [21] and ranged from 123 to 168 mg GAE/g dry matter. In our study, the lowest values of antioxidant activity of white grapevine varieties were found in the Welschriesling variety—8374 µg/g in year 2015; the higher values in grape seeds of white grapevine varieties were found in Gewürzetraminer—12,050 µg/g in year 2017.

Using three different methods (FRAP, DPPH, and ABTS), the antioxidant activity and the content of polyphenolic compounds in Çalkarası grape seeds obtained from a Turkish winery were determined. Antioxidant activity was expressed in µmol Trolox/g dry matter. The value of antioxidant activity was 19.96 µmol TE/g for the FRAP method, 19.30 µmol TE/g for the DPPH method, and 16.45 µmol TE/g for the ABTS method. The content of total polyphenolic compounds was 27.92 mg GAE/g dry matter [24].

In our experiments, the average value of antioxidant activity of grapevine seeds determined by the DPPH method reached 10,628 µg/g GAE. It was 13,583 µg/g GAE when measured by the FRAP method; 4972 µg/g GAE with the ABTS method and 2574 µg/g GAE with the FR method. The average value of the content of total polyphenolic compounds was 8803 µg/g GAE.

The values found in the studies, which are focused on the determination of antioxidant components, differ considerably from each other. The reason for this is that each of the studies used a different methodology of extraction procedures. Thus, the varieties were macerated at different times, with different solvents (methanol, ethanol, acetone), with a different seed-to-supernatant dilution ratio, and with different sample drying procedures, etc. Of course, the variations are also due to different collection years, varieties, seed ripening times, and collection areas, etc. In terms of conversions, there are differences related to expression in other units (liters versus kilograms) as well as differences between dried and fresh samples. Last but not least, there were variances due to the use of different analytical methods. Due to such different testing methods, any comparison between studies is not relevant. However, what can be established from the studies is that, to a large extent, catechin and epicatechin are abundant compounds. This information corresponds to our study, in which epicatechin was the most common component.

In addition, the methods for determining antioxidant activity and standards were different; specifically, the antioxidant activity expressed in (1) equivalents of different standards (most often equivalents of gallic acid, Trolox, or catechin), (2) relative percentage, and (3) time loss of absorbance. In addition, the expression of antioxidant activity values changes over time as the radicals for determining antioxidant activity are highly reactive [25].

The differences between the individual studies are surprising. At the end of the discussion, we can say that our results were in the range of values given by other studies. None of our results showed extreme deviations from the results of other studies.

## 4. Material and Methods

### 4.1. Biological Material and Chemicals

Experimental material—vine seeds (*Vitis vinifera* L.)—was obtained from varieties grown around the Institute of Viticulture and Enology (Mendel University), Czech Republic. These varieties were Cerason, Laurot, Kofranka, Gewürztraminer, Hibernal, Blaufrankisch, Zweigeltrebe, Erilon, Palava, and Welschriesling. This biological material was obtained from the years 2015, 2016, and 2017.

### 4.2. Sample Preparation

A separation machine was used to separate the *Vitis vinifera* L. seeds from the pulp; these were then cleaned, dried, and ground. The separated seeds were washed, and any empty seeds were removed. The washed seeds were dried in an oven at 50°C for 12 h and subsequently ground using a coffee grinder (BOSCH MKM6003).

The ground seeds were extracted in 75% ethanol for 120 h, in a ratio of 1 part ground seeds to 10 parts ethanol (*w*/*w*). After this time, the extract was transferred into 2 mL microtubes (Eppendorf, Hamburg, Germany) and centrifuged (MiniSpin Centrifuge, Eppendorf, Hamburg, Germany). The supernatant of centrifuged sample was separated into the another microtube, and thus prepared was taken for spectrometric and chromatographic analysis.

### 4.3. Chemicals

The following chemicals were used: ethanol 96%, deionized water, stable free radical 2,2-diphenyl-1-picrylhydrazyl (DPPH), radical cation 2,2´-azinobis 3-ethylbenzothiazoline-6-sulfonic acid (ABTS), methanol 96%, acetic acid 0.2%, liquid nitrogen, 2,4,6-tripyridyl-s-triazine (TPTZ), hydrochloric acid (HCl), ferric chloride (FeCl_3_), acetate buffer, sodium acetate, Folin–Ciocalteu reagent, and sodium carbonate (Na_2_CO_3_) decahydrate. Chemicals from Sigma-Aldrich (Germany) were used for the experiments.

### 4.4. Determination of Antioxidant Activity

In order to ensure the objectivity of the results, four fundamentally different methods were used to determine the antioxidant activity. Samples were analyzed on a BS-400 automatic spectrophotometer (Mindray, Shenzen, China). All samples were analyzed in triplicate, the resulting value corresponding to the average of these measurements.

#### 4.4.1. Determination of Antioxidant Activity Using the ABTS Method

A solution for the determination of antioxidant activity by ABTS assay was prepared by mixing two solutions—solution 1: 7 mM solution of ABTS prepared by weighing m = 9.60 mg per 5 mL of distilled water; and solution 2: 4.95 mM solution of potassium persulfate prepared by weighing m = 1.67 mg per 5 mL of distilled water. The resulting solution was diluted 1:10 with distilled water and left in the dark and cold for 12 h.

150 µL of reagent R1 (7 mM ABTS and 4.95 mM potassium persulfate) was pipetted into plastic cuvettes, followed by the addition of 3 µL of sample and measured in spectrophotometer (λ = 660 nm) for 12 min. According to the calibration curve, the absorbance was converted to the equivalent content of gallic acid; the antioxidant activity was calculated from the calibration curve using gallic acid as a standard (10–200 mg∙L^−1^), and the results were expressed as gallic acid equivalents.

#### 4.4.2. Determination of Antioxidant Activity Using the DPPH Method

M = 9.35 mg of radical DPPH was weighed. This amount was transferred to a 250 mL volumetric flask and made up with methanol.

150 µL of reagent R1 (0.095 mM DPPH) were pipetted into plastic cuvettes, followed by the addition of 15 µL of sample to be measured. The DPPH test is based on the ability of the stable free radical 2,2-diphenyl-1-picrylhydrazyl to react with hydrogen donors. DPPH shows strong absorption in the ultraviolet-visible spectroscopy (UV-VIS) spectrum. Absorbance was measured for 12 minutes at λ = 505 nm. According to the calibration curve, the absorbance was converted to an equivalent gallic acid content.

#### 4.4.3. Determination of Antioxidant Activity Using the Ferric Reducing Antioxidant Power Method

Three solutions were used to determine the antioxidant activity using the ferric reducing antioxidant power (FRAP) method—(1) TPTZ solution: 10 mM TPTZ (m = 78.02 mg) dissolved in 25 mL of 40 mM HCl; (2) FeCl_3_ solution: 20 mM FeCl_3_ (m = 135.13 mg) dissolved in 25 mL of distilled water; and (3) acetate buffer solution: 0.02 M acetate buffer pH 3.6 (m = 775 mg sodium acetate dissolved in 250 mL of distilled water, pH adjusted with acetic acid). The three solutions were mixed in the ratio 1:1:10 (TPTZ:FeCl_3_:acetate buffer).

150 µL of reagent were pipetted into plastic cuvettes and then 3 µL of sample were added. Absorbance was measured for 12 minutes at λ = 605 nm. Antioxidant activity was calculated from the calibration curve using gallic acid as a standard (10–200 mg∙L^-1^). Results were expressed as gallic acid equivalent.

#### 4.4.4. Determination of Antioxidant Activity Using the Chlorophyllin Free Radical Method

200 µL of reagent R1 (0.1 M HCl, chlorophyll extract, reaction buffer, catalyst) were pipetted into plastic cuvettes and then 8 µL of sample were added. This method uses the ability of chlorophyllin (sodium-copper salt of chlorophyll) to accept and donate electrons while stably changing the absorption maximum. This process is conditioned by the alkaline environment and the addition of a catalyst. Absorbance was measured for 12 min at λ = 450 nm. The last measurement values were used for the calculation. Results were expressed as gallic acid equivalent.

### 4.5. Determination of Total Polyphenol Concentration

The Folin–Ciocalteu method was used to determine total polyphenolic compounds. All samples were analyzed in triplicate, and the resulting value was obtained as the average of these measurements.

A 40 µL sample was pipetted into a cuvette (3 mL) and diluted with 1960 µL of distilled water. Subsequently, 50 µL of Folin–Ciocalteu reagent was added to the cuvette. The mixture was shaken thoroughly and, after 3 minutes, 300 µL of 20% Na_2_CO_3_ decahydrate solution were added. The reaction mixture was shaken and incubated at 22°C for 120 minutes. After this time, the absorbance (SPECORD 210, Carl-Zeiss, Jena, Germany) was measured at λ = 750 nm against a blank. Results were expressed as gallic acid equivalent.

### 4.6. Determination of Individual Antioxidant Components Using High-Performance Liquid Chromatography with Ultraviolet-Visible Spectroscopy

Determination of selected antioxidant components using high-performance liquid chromatography (HPLC) with UV/VIS was performed by direct sample injection method. The prepared samples were diluted 10x with 100 mM perchloric acid (HClO_4_) and then used for HPLC analysis.

The following were used in the study: instrumentation—Shimadzu LC-10A binary high-pressure system; controller system—SCL-10Avp; 2 pumps—LC-10ADvp, column thermostat with manual injection valve; rheodyne—CTO-10ACvp; DAD—SPD-M10Avp; software—LCsolution. The separation was performed on an Alltech Alltima HP C18 3 μm column; 3 × 150 mm at 50°C. The injection volume of the sample was 20 μL, the flow rate of the mobile phase was set at 0.9 mL.min^−1^. The composition of mobile phase A was 15 mM HClO_4_ and of mobile phase B was 15 mM HClO_4_ and 80% acetonitrile.

The gradient program was: 0.00 min—3% B; 3.00 min—6% B; 15.00 min—24% B; 18.00 min—30% B; 19.50 min—36% B; 21.00 min—48% B; 21.50 min—60% B; 22.00 min—60% B; 22.01 min—0% B; 23.99 min—0% B; and 24.00 min—3% B. The total analysis time was 27 min. Data in the range of 200–520 nm were recorded for 24 min. The determination of individual components was performed on the basis of calibration standards. The following compounds were determined: gallic acid, caffeic acid, coumaric acid, coutaric acid, ferulic acid, fertaric acid, trans-piceid, trans-piceatannol, rutin, quercetin-3-β-D-glucoside, quercitrin, myricetin, catechin, and epicatechin.

### 4.7. Statistical Analysis

All measurements were performed in triplicates. Statistical analyses were generated using Excel 2007 software packages (manufactured by Microsoft Office, Redmond, WA, USA) and Statistica 10 statistical software (Copyright © StatSoft). Analysis of variance (ANOVA) was used to verify the differences between the variants of experiment, specifically using the nonparametric Kruskal–Wallis test. Pearson’s correlation coefficient was used to determine the correlations between the variants.

## 5. Conclusions

Grape seeds are rich in antioxidants. Evidence for this claim includes many scientific studies pointing to their antioxidant potential.

The average value of antioxidant activity of grapevine seeds determined by the DPPH method was 10,628 µg/g GAE. It was 13,583 µg/g GAE when measured by the FRAP method; 4972 µg/g GAE by the ABTS method; and 2574 µg/g GAE by the FR method. The average value of the content of total polyphenolic compounds was 8803 µg/g GAE. The highest values of antioxidant activity were achieved by the Cerason variety; the lowest values were measured in the Riesling variety. The highest values of antioxidant activity and content of total polyphenolic compounds were reached in 2017; the lowest values were reached in 2015. Seeds from blue varieties showed higher values than seeds from white varieties.

The content of 14 phenolic compounds (gallic acid, caffeic acid, coumaric acid, coutaric acid, ferulic acid, fertaric acid, trans-piceid, trans-piceatannol, rutin, quercetin-3-β-D-glucoside, quercitrin, myricetin, catechin, and epicatechin) was determined by HPLC/UV-VIS. The most represented compound was gallic acid, with an average content of 225.4 µg/g in all monitored varieties. It was most present in the Cerason variety (298 µg/g) and least present in the Palava variety (160 µg/g). In contrast, the least represented compound was trans-piceatannol, which had an average content of 0.326 µg/g. It was most present in the Kofranka variety (0.51 µg/g); the lowest content was recorded in the Palava variety (0.22 µg/g).

The data obtained in this study are an important indicator of the potential nutraceutical and economic utility of this waste material in the future. Among other things, these results provide useful information for real potential use in the different branches of the food industry.

## Figures and Tables

**Table 1:** Pearson correlation coefficient between two methods for determination of antioxidant activity and concentration of total polyphenols.

**2015**				
	FRAP	ABTS	CHFR	TP
DPPH	0.97	0.95	0.95	0.95
FRAP	x	0.96	0.95	0.94
ABTS	x	x	0.93	0.93
CHFR	x	x	x	0.92
**2016**				
	FRAP	ABTS	CHFR	TP
DPPH	0.79	0.88	0.87	0.80
FRAP	x	0.83	0.84	0.89
ABTS	x	x	0.97	0.95
CHFR	x	x	x	0.94
**2017**				
	FRAP	ABTS	CHFR	TP
DPPH	0.86	0.92	0.91	0.93
FRAP	x	0.89	0.84	0.79
ABTS	x	x	0.87	0.90
CHFR	x	x	x	0.92

**Table 2:** Average concentration of analyzed compounds in 2015. The results are expressed in µg/g of dry matter.

**Chemical Compound**	**Gallic Acid**	**Caffeic Acid**	**Coumaric Acid**	**Coutaric Acid**	**Ferulic Acid**	**Fertaric Acid**	**Catechin**
Average concentration	225.4	27.92	1.76	5.51	3.10	4.24	181.3
**Chemical Compound**	**Epicatechin**	**Trans-Piceid**	**Trans-Piceatannol**	**Rutin**	**Quercetin-3-β-D-Glucoside**	**Quercitrin**	**Myricetin**
Average concentration	138.2	4.69	10.15	0.33	5.60	1.92	1.84

**Table 3:** Average content of selected antioxidant components in the Cerason variety in 2015–2017. The results are expressed in µg/g of dry matter.

**Chemical Compound**	**Gallic Acid**	**Caffeic Acid**	**Coumaric Acid**	**Coutaric Acid**	**Ferulic Acid**	**Fertaric Acid**	**Catechin**
Average concentration	333	32	2.3	7.4	4.7	4.9	182
**Chemical Compound**	**Epicatechin**	**Trans-Piceid**	**Trans-Piceatannol**	**Rutin**	**Quercetin-3-β-D-Glucoside**	**Quercitrin**	**Myricetin**
Average concentration	167.5	5.4	10.9	0.34	14.8	6.1	3
